# Differences in and drivers of mental, social, functional, and financial well-being during COVID-19: Evidence from Australia, France, Germany, and South Africa

**DOI:** 10.1371/journal.pone.0276077

**Published:** 2022-10-13

**Authors:** Arvid Hoffmann, Daria Plotkina, Marie-Hélène Broihanne, Anja Göritz, Stefanie Kleimeier

**Affiliations:** 1 Adelaide Business School, University of Adelaide, Adelaide, Australia; 2 EM Strasbourg Business School, University of Strasbourg, Strasbourg, France; 3 Department of Psychology, University of Freiburg, Freiburg, Germany; 4 Faculty of Management, Open University, Heerlen, The Netherlands; 5 School of Business and Economics, Maastricht University, Maastricht, The Netherlands; 6 University of Stellenbosch Business School, University of Stellenbosch, Cape Town, South Africa; Wollo University, ETHIOPIA

## Abstract

COVID-19 has a substantial and unexpected impact on individuals’ daily life around the world. Unprecedented public health restrictions such as lockdowns have the potential to affect multiple dimensions of individuals’ well-being, while the severity of such restrictions varies across countries. However, a holistic perspective comparing differences in and drivers of the different dimensions of well-being across countries differentially affected by COVID-19 is missing to date. We address this gap in the literature by examining the mental, social, functional, and financial well-being of 2,100 individuals across Australia, France, Germany, and South Africa by means of a survey administered during May of 2021. Supporting our holistic approach, we find that the different dimensions of well-being are correlated, with survey respondents from France reporting the lowest and those from Australia reporting the highest overall level of well-being. Respondents’ subjective and objective evaluations of their living conditions during lockdowns as well as positive health and financial behaviors are positively associated with their well-being during the pandemic.

## Introduction

The year 2020 struck with an unsurpassed health and economic crisis: the COVID-19 outbreak. The pandemic resulted in unprecedentedly strict government policies affecting individuals’ daily life [1]. While health considerations were central in governmental decision-making around lockdowns and social distancing at the start of the pandemic, financial and social considerations gained prominence later on [2]. Indeed, while for some the pandemic had health consequences in terms of illness or medical expenses, for others the virus had financial consequences in terms of job loss or reduced income. For yet another group, the virus had both health and financial consequences, for example, when a family’s main income earner died of COVID-19. In this regard, the interrelation of finance and health aspects can create a vicious circle, as financial stress can be both a cause and consequence of poor health [3, 4]. Furthermore, lockdowns and other restrictive policy measures also represent risks to individuals’ mental and social well-being [5].

Prior research shows that individuals’ physical, mental, and financial health are linked [6] and that a sudden loss of income can have a devastating impact on one’s health and well-being [7]. While there has been substantial interest in differences in individual well-being during the COVID-19 pandemic [e.g., 8–10], to date we have only a limited understanding of issues such as: (i) the interrelation of the different dimensions of individual well-being, (ii) the variation across countries in terms of how individual well-being is affected by the pandemic, and (iii) the drivers of the different dimensions of individual well-being within countries during the pandemic [e.g., 11–13].

We address this gap in the literature and assess the mental, social, functional, and financial well-being of 2,100 individuals across four countries from three continents: Australia, France, Germany, and South Africa. Importantly, at the time of data collection (we conducted a survey during May of 2021) these countries had very different COVID-19 case numbers, death rates, and public health restrictions. Moreover, the countries under investigation allow us to explore the experience of individuals across a variety of cultures, climates, levels of economic development, and opportunities for enforcing border restrictions (e.g., in contrast to the other countries we study, Australia as an island state was able to seal itself off from the rest of the world during the pandemic).

The timing of our survey means that we capture well-being among individuals who have had the chance to accumulate personal experience with the pandemic and understand how it affects their daily life. Hence, we can investigate the impact of directly experiencing COVID-19 and relate differences in this impact across countries to variations in government restrictions and support for affected individuals. Furthermore, the timing of our survey means that the first emotional and/or anxious reactions of individuals likely have passed (and the virus is better known) which helps us to better capture well-being. Finally, we are able to relate differences in individual well-being to government restrictions since people had a longer experience of them.

The contribution of our study lies in providing a holistic snapshot of individual well-being across countries differentially affected by COVID-19. We also contribute by accounting for both objective indicators and subjective evaluations of living conditions during lockdowns [14, 15] as well as positive health [16, 17] and financial behaviors [18], as prior studies stress the importance of where people live and their daily habits for their well-being during crises. Finally, we contribute to the literature by being the first to address the link between experiencing COVID-19 first-hand and the different dimensions of individual well-being while at the same time examining the interrelation of these dimensions both within and across countries. Our research builds on the emerging literature on the consequences of COVID-19 for individuals. According to the World Health Organization [2], managing one’s mental health and psycho-social well-being during the pandemic is as important as managing one’s physical health at a time when these dimensions of well-being are at risk due to the increased stress and restrictions associated with COVID-19.

## Theoretical background and hypotheses

Individual well-being is generally measured by subjective well-being, comprising affective and cognitive evaluations of one’s life. Ryan and Deci [19] posit that well-being should be approached and measured as a multi-dimensional concept. Building on this logic, we follow Halleröd and Seldén [20] and differentiate between physical, mental, social, functional, and financial well-being during the COVID-19 pandemic. Physical well-being can be captured by acute or chronic physical illness. We follow Dryhurst et al.’s [21] work on individuals’ experience with COVID-19 and conceptualize it as one’s actual experience with the virus (i.e., being diagnosed with the virus, suspecting having contracted it, or having a relative diagnosed with it). We include confirmed as well as suspected infection in our conceptualization of physical well-being given that already the fear of being infected affects individuals’ subjective well-being [22]. Mental or psychological well-being is related to individuals’ mental states and perceptions such as anxiety, worry, and stress. We follow Barrett, Hogreve [23] and rely on Tennant et al.’s [24] conceptualization of mental well-being. Social well-being captures people’s fulfilment in terms of social interactions and sense of belonging to a community [20]. It evaluates the creation and maintenance of high-quality social interactions and relationships. Functional well-being describes to which extent people can carry out intended activities [20]. Finally, financial well-being is conceptualized as consisting of two dimensions: current money management stress and expected future financial security [25].

To guide our empirical analysis, we develop a series of exploratory hypotheses based on findings from the emerging research on COVID-19 and individual well-being, thus following “the scientific method” of first developing hypotheses, which are then tested empirically [26]. In particular, the following exploratory hypotheses will be tested using the collected survey data:

First (H1), we expect cross-country differences in the dimensions of individual well-being based on how severely countries were impacted by COVID-19 and the variation in public health restrictions and support packages (Tables 2 and 4).

Second (H2), negative shocks to one of the dimensions of individual well-being can create a vicious cycle and negatively affect another dimension of individual well-being [20, 27]. We therefore expect the scores reported by survey respondents for the different well-being dimensions to be correlated.

Third (H3), given previously discovered negative effects of COVID-19 on the different dimensions of individual well-being [23, 28] we also expect that a direct COVID-19 experience is negatively associated with individual well-being during the pandemic.

Fourth (H4), the pandemic has shown that not all individuals face the same challenges. For example, women [29], younger individuals [30], and unemployed or low-income individuals [31] are more at risk of low well-being. Finally, one’s country of residence [30] and ethnicity [32] are related to mental and physical well-being during the pandemic. We thus expect that socio-demographic factors are associated with differences in individual well-being during the pandemic.

Fifth (H5), we expect that objective living conditions are associated with individual well-being during the pandemic. Aspects such as household size [14], area of residence and presence of an outdoor space [15, 33], relationship status [34], and number of dependents [35] can all affect individual well-being. Lockdowns stressed the importance of living in a low-density neighborhood, having enough indoor space, and access to outdoor space to sustain one’s well-being [36, 37].

Sixth (H6), as well-being is a subjective evaluation of one’s state, we expect that beyond aforementioned objective living conditions during the pandemic, the subjective evaluation of these living conditions is also important. Indeed, the pandemic stressed the importance of satisfaction with one’s housing comfort [38]. Although objective factors can explain discomfort or dissatisfaction of individuals with their living conditions, the subjective evaluation thereof is the main explanatory variable of individual happiness, mental, physical, and overall well-being [36, 39].

Seventh (H7), one’s own behavior as an individual during the pandemic can also affect individual well-being. Positive health [16, 17] and financial behaviors [18, 40] are expected to be positively associated with individual well-being. For example, physical exercise improves mental and overall well-being [30, 41, 42]. Moreover, healthy eating is positively related to individual well-being [43], while binge-drinking is negatively related to individual well-being, especially during the pandemic [44]. Finally, negative financial behaviors such as gambling are a risk to individual well-being, especially given its alarming increase during the pandemic [45].

## Data and method

### Data collection

We conducted a survey in Australia, France, Germany, and South Africa in May of 2021. Respondents were recruited from an online panel maintained by Qualtrics, using quotas to represent as closely as possible national populations in terms of gender, age, income, and ethnicity. Doing so, non-probability voluntary sampling was employed to make our questionnaire available to potential respondents. Respondents were informed that completion of the survey implied consent to use their anonymous data, and no personal details allowing identification of respondents were requested. As per the guidelines of the first author’s university, research for which there is no foreseeable risk of harm or discomfort; and any foreseeable risk is no more than inconvenience; and that involves the use of records that contain only non-identifiable data about human beings is exempt from ethics review. According to study power calculations, we needed a minimum of 385 respondents per country sample. Considering this threshold and the available funding, we aimed to recruit approximately 500 respondents per country. The final sample consisted of 494 respondents from Australia, 549 from France, 510 from Germany, and 547 from South Africa, for a total of 2,100 respondents completing the survey and providing a valid response in terms of meeting standard data quality requirements such as not providing the same answer for each question or highly implausible answers.

Individual country samples approximate their respective national statistics in terms of socio-demographics (Table 1) and COVID-19 statistics (Table 2). Exceptions to this overall pattern are an oversampling of affluent individuals in Australia and South Africa, and an oversampling of individuals suspecting to have been infected with COVID-19 but not having been formally diagnosed and those reporting COVID-19 infections within the household in South Africa. The latter pattern might be explained by the lower accessibility of diagnostics and larger households in this country.

**Table 1:** Comparison of National statistics with the study sample.

	Australia		France		Germany		South Africa	
	Study Sample	National Average	Study Sample	National Average	Study Sample	National Average	Study Sample	National Average
**Average age** ^**1**^	46.88	37.3	49.86	42.3	50.28	45.7	37	27.6^1^
**Gender** (male)	51%	49.6%	47.5%	48.4%	50%	49%	51%	49.3%
**Average gross annual income**	$ 62,605	$39,506	$ 24,041	$39,478	$34,890	$44,578	$12,658	$4,091
**Ethnicity** (% of total population)	White– 79.8%	White– 89.9%	White– 92.9%	White^2^ – 85%	White– 92.5%	White^3^ – 88.2%	White– 32.9%	White– 6.6%
	Asian / Indian –14.6%	Aboriginal –3.2%	Black African– 1.8%	Middle-Eastern– 10%	Asian– 1.2%	Middle-Eastern –2.8%	Black– 51.2%	African– 82.2%
	Aboriginal– 1.9%		Middle-Eastern –1.4%	Black African– 3.5%	Black African 0.4%		Colored– 8%	Colored– 8.7%
							Asian– 6.9%	Indian / Asian– 2.5%

All statistics from Euromonitor (Passport) for 2020 if not indicated otherwise.

^1^ https://www.worldometers.info

^2^ https://worldpopulationreview.com/

^3^ https://www.diversityabroad.com/articles/travel-guide/germany

**Table 2:** National and study sample statistics of COVID-19 during the sample period.

	Australia	France	Germany	South Africa
	**National Statistics** ^**1**^			
**Confirmed total COVID-19 cases by May 31, 2021** (number of individuals)	30,106	5,667,324	3,689,918	1,665,617
**Confirmed COVID-19 cases** (% of population)	0.11%	8.68%	4.40%	2.80%
**Deaths cases from COVID-19 by May 31, 2021** (number of individuals)	910	109,533	89,471	56,506
	**Study Sample**			
**Confirmed COVID-19 cases** (% of sample)	2.2%	6%	3.1%	5.7%
**Not confirmed COVID-19, presence of symptoms** (% of sample)	19.8%	19.7%	19.2%	32.9%
**Confirmed COVID-19 cases within household** (% of sample)	3.8%	10.4%	6.9%	25.2%
**COVID-19 experience** (% of sample)^2^	23.1%	30.4%	24.7%	48.1%

^1^Source is https://www.worldometers.info/coronavirus/.

^2^The statistic shows the percentage of individuals that have either confirmed or not confirmed COVID-19 experience or a case of COVID-19 within their household; the change relates to the difference between May 1, 2021 and May 31, 2021.

### Survey measures

In our analysis of the differences in and drivers of individual well-being during the COVID-19 pandemic, we distinguish the following groups of explanatory variables: having had a COVID-19 experience; socio-demographics; objective indicators of living conditions during lockdowns; subjective evaluation of living conditions during lockdowns; positive health behaviors; positive financial behaviors. We discuss the indicators of each group of explanatory variables next, followed by a discussion of our measures of the different dimensions of individual well-being.

#### COVID-19 experience

To measure a direct COVID-19 experience and account for physical well-being, we asked respondents whether since February 2020 they or someone in their household have had any symptoms or signs of illness that made them belief to have contracted COVID-19. We also asked whether the COVID-19 infection was confirmed by a test. We focus on the subjective perception of having had COVID-19 instead of only confirmed cases, since already the fear of being infected affects individuals’ subjective well-being [22].

#### Socio-demographics

To measure respondents’ socio-demographic characteristics, we asked standard questions on age, education, employment, ethnicity, gender, and income. These measures were inspired by previous research on individual differences in well-being during the pandemic [e.g., 29, 30, 32].

#### Objective indicators of living conditions during lockdowns

To evaluate respondents’ objective living conditions during lockdowns, they were asked to share details on their area of residence (i.e., urban vs. rural), presence of an external space (e.g., balcony, terrace, garden), size of indoor space, number of dependents in the household (e.g., children, disabled, elderly) and relationship status. These questions were based on a survey by the National Agency of Public Health in France that evaluated individuals’ living conditions during the pandemic [46].

#### Subjective evaluation of living conditions during lockdowns

Respondents’ subjective evaluation of living conditions during lockdowns was measured by asking “How do you evaluate your living conditions with regard to COVID-19-related stay-at-home restrictions?” This measure was adopted from aforementioned survey by the National Agency of Public Health in France.

#### Positive health behaviors

Positive health behaviors during COVID-19 were measured on a seven-item formative scale as previously used by Hoffmann and Risse [6] based on the Alameda 7 Index of healthy habits [47], with items such as “I eat breakfast 7 days per week”, “I exercise more than 3 days per week”, “I avoided binge-drinking during past year.”

#### Positive financial behaviors

Positive financial behaviors during COVID-19 were also measured using a nine-item formative scale taken from Hoffmann and Risse [6] who based their measure on Hilgert, Hogarth [48], with items such as “My household has no outstanding bills”, “I save regularly by putting money aside”, “I do not participate in any type of gambling.”

#### Individual well-being

Respondents evaluated their individual well-being according to well-established scales (see Table 3 for details). Mental well-being was measured with nine items of the Warwick-Edinburgh Mental Well-Being Scale (WEMWBS) from Tennant, Hiller [24]. Social well-being was evaluated with five items from the Mental Health Continuum-Short Form (MHC-SF) scale from Lamers, Westerhof [49] based on the Mental Health Continuum of Keyes [50]. Functional well-being was gauged with three items from Barrett, Hogreve [23]. Financial well-being was assessed as in Netemeyer, Warmath [25], with five items each for expected future financial security and current money management stress. All well-being scales used seven-point Likert scale for consistency and uniform appearance, and have Cronbach’s alpha [51] and composite reliability [52] exceeding 0.70. The only exception is the functional well-being scale, which did not meet the cut-off value for Cronbach’s alpha but did have satisfactory composite reliability. Given that this is a well-established scale, we did not remove any items to improve Cronbach’s alpha. Confirming convergent validity, all items load significantly only on their underlying construct and the average variance extracted (AVE) exceeds 0.50 [53]. To establish discriminant validity, we verify that the intercorrelations between latent factors do not include unity [54] while each construct’s AVE is greater than the squared correlations between any set of two constructs [53].

**Table 3:** Scale items, factor loadings, and construct validity of individual well-being.

Scale and Authors	Items	Mean	SD	Factor Loading	Cronbach’s Alpha	CR	AVE
**Mental well-being** (Tennant et al., 2007)	1. I have been feeling useful	4.34	1.62	.711	.908	.925	.582
	2. I have been feeling relaxed	4.47	1.55	.772			
	3. I have been feeling interested in other people	4.44	1.58	.630			
	4. I have had energy to spare	4.33	1.59	.677			
	5. I have been dealing with problems well	4.74	1.49	.792			
	6. I have been thinking clearly	5.03	1.51	.807			
	7. I have been feeling good about myself	4.69	1.62	.856			
	8. I have been feeling close to other people	4.46	1.65	.733			
	9. I have been feeling confident	4.54	1.64	.856			
**Social well-being** (Lamers et al., 2011)	1. I have something important to contribute to society	4.03	1.69	.681	.823	.879	.594
	2. People are basically good	4.10	1.49	.772			
	3. I belong to a community (like a social group, club, neighborhood)	3.68	1.92	.727			
	4. Our society is becoming a better place for people	3.34	1.59	.858			
	5. The way our society works makes sense to me	3.53	1.60	.805			
**Functional well-being** (Barrett et al., 2021 from Halleröd & Seldén, 2013)	1. During the pandemic, I have been able to perform my daily activities as usual	4.06	1.90	.605	.555	.771	.534
	2. During the pandemic, I have felt restrained in my mobility (R)	3.46	1.94	.844			
	3. I have perceived the public health measures related to COVID-19-19 in the area that I live in as restrictive (R)	3.82	1.81	.724			
**Financial well-being: Expected financial security** (Netemeyer et al., 2018)	1. I am becoming financially secure	3.96	1.79	.833	.913	.934	.742
	2. I will be financially secure until the end of my life	3.91	1.86	.883			
	3. I have saved (or will be able to save) enough money to last me to the end of my life	3.50	1.96	.859			
	4. I am securing my financial future	4.16	1.85	.884			
	5. I will achieve financial goals that I have set for myself	4.31	1.80	.847			
**Financial well-being: Current money management stress** (Netemeyer et al., 2018)	1. Because of my money situation, I feel I will never have the things I want in life	3.74	1.92	.674	.839	.886	.611
	2. I am behind with my finances	3.16	1.93	.777			
	3. My finances control my life	3.64	1.86	.812			
	4. Whenever I feel in control of my finances, something happens that sets me back	3.68	1.88	.840			
	5. I am unable to enjoy life because I obsess too much about money	3.02	1.81	.797			

SD = Standard deviation; CR = Composite Reliability; AVE = Average Variance Extracted; (R) = reverse-scored item.

### Common method variance

As all our measures are survey-based, common method variance (CMV) could be a concern. To minimize this risk, we included reverse-coded items to minimize compliance effects [55]. We also verified that the variables do not load on a single factor in a Harman’s single-factor test with exploratory factor analysis [56]. Finally, we included a theoretically unrelated question on attitude toward the color blue [57] and conduct a Lindell and Whitney [55] marker variable test. The marker variable does not correlate significantly with any of the other variables. In sum, all tests indicate CMV risk is not a concern.

### Country-specific COVID-19 policies

The studied countries differ in COVID-19 related policy measures and public health restrictions at the time of the survey (see Table 4). For example, while Australian citizens had largely free movement within the country, no curfew, and could gather with over 10 people, French and German citizens only began to exit from national lockdown restrictions. COVID-19 restrictions could be ranked from most to least stringent as follows: France, Germany, South Africa, and Australia. Finally, all countries started to propose mental support and vaccination of vulnerable individuals.

**Table 4:** Country-specific COVID-19 related policy measures and health restrictions during the sample period.

Country/ COVID-19 Related Restriction	Australia ^1^	France ^2^	Germany ^3^	South Africa ^4^
**Level of restrictions**	low	High	medium	low-medium
**Entry to country allowed**			✓ for vaccinated	✓
**Free movement within country allowed**	✓		✓	✓
**Curfew in place**		✓		✓
**Free to leave one’s home**	✓	✓	✓	✓
**Schools open**	✓	✓		✓
**Cultural sites open**	✓	✓	✓ (+special fund)	✓
**Hospitality open**	✓	outdoor	✓ (end of May 2021)	✓
**Working from home only**	✓	✓	✓	✓
**Gathering of > 10 people allowed**	✓		✓ (if fully vaccinated)	✓
**Mental support provided**	✓	✓	✓	✓
**Nationwide state of emergency**	✓	till June 30, 2021	till June 30, 2021	✓ (Level 1 till May 30, 2021; Level 2 after)
**Stringency legacy index** ^**5**^	55/100	75/100	79/100	70/100
**Government response index** ^**6**^	47/100	72/100	67/100	56/100

✓ = policy measure or health restriction is present.

Sources

^1^https://www.reuters.com/world/asia-pacific/australia-bans-arrivals-india-says-offenders-face-jail-fines-2021-05-01/; ^2^https://www.rfi.fr/en/france/20210430-timeline-for-lifting-covid-restrictions-in-france-in-may-june-emmanuel-macron-economy-curfew-lockdown

^3^https://www.deutschland.de/en/news/german-federal-government-informs-about-the-corona-crisis; ^4^https://www.gov.za/covid-19/resources/regulations-and-guidelines-coronavirus-covid-19.

^5^https://ourworldindata.org/: this index is a composite measure based on nine response indicators including the policy indicators such as school and workplace closures, staying at home policies, mask obligations, cancelation of public events and gatherings, international travel controls, testing policies, contact tracing, vaccination policy.

^6^https://ourworldindata.org/: this index is a composite measure based on the response indicators from containment and closure policies; economic policies; health system policies; vaccination policies.

## Results

### Between-country comparison of overall differences in individual well-being

**Fig 1:**
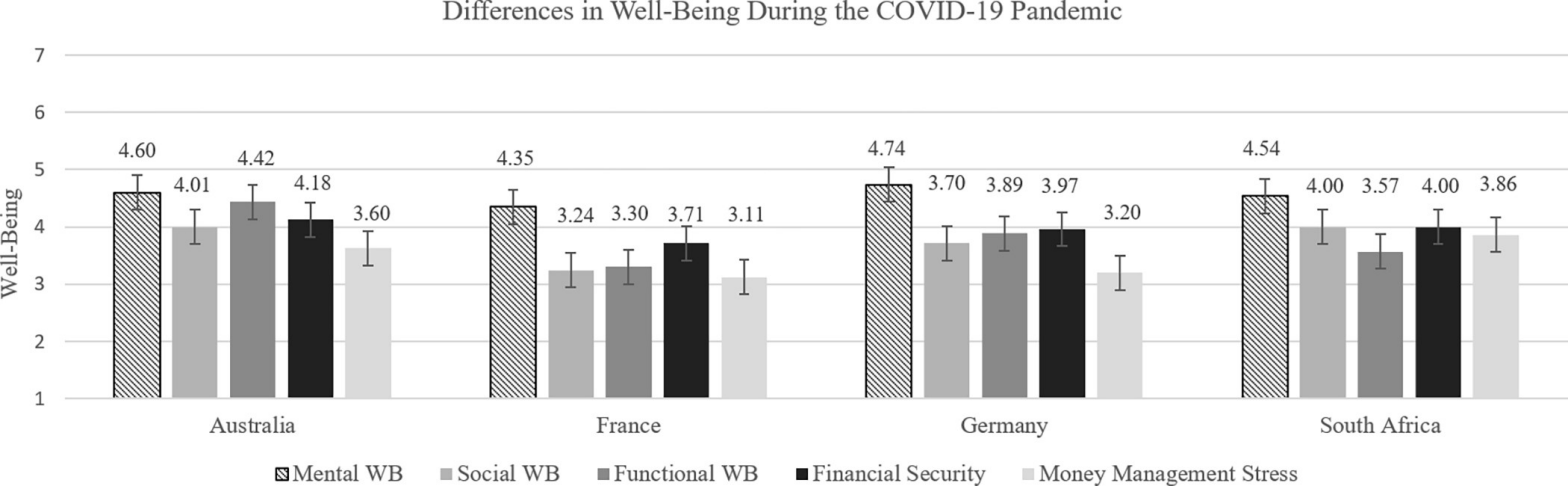
Differences in survey respondents’ individual well-being across Australia, France, Germany and South Africa during the COVID-19 pandemic. This figure presents the means of mental, social, functional, and financial well-being (expected financial security and current money management stress) of survey respondents during the COVID-19 pandemic as of May 2021. The scale ranges from 1 = low well-being to 7 = high well-being. The bars show 5% confidence intervals.

Fig 1 presents descriptive results showing respective levels of individual well-being of the survey respondents across all dimensions and in each of the four countries that we study, while Table 5 provides means, standard deviations, and differences in levels of well-being among these countries. Unless noted otherwise, all results that we discuss below are significant at the 5% level or better.

**Table 5:** Differences in survey respondents’ well-being across Australia, France, Germany and South Africa during the COVID-19 pandemic.

	Australia		France		Germany		South Africa	
	Mean	SD	Mean	SD	Mean	SD	Mean	SD
**Mental Well-Being**	4.60^z^	1.19	4.35^x,y,z^	1.14	4.74^w,y^	1.09	4.54^w,x^	1.34
**Social Well-Being**	4.01^y,z^	1.25	3.24^w,z^	1.17	3.70^v,x,y^	1.13	4.00^v,w,x^	1.36
**Functional Well-Being**	4.42^x,y,z^	1.23	3.30^v,w,z^	1.36	3.89^u,w,y^	1.45	3.57^u,v,x^	1.17
**Financial Well-Being: Expected Financial Security**	4.18^z^	1.61	3.71^z,y^	1.50	3.97	1.53	4.00^y^	1.70
**Financial Well-Being: Current Money Management Stress**	3.60^y,z^	1.54	3.11^x,z^	1.24	3.20^y^	1.47	3.86^x^	1.49

This table presents the means and standard deviations (SD) of mental, social, functional, and financial well-being (expected financial security and current money management stress) of individuals during the COVID-19 pandemic as of May 2021. Cells sharing the same superscript are significantly different from each other at the 5% significance level.

Overall, survey respondents’ mental well-being is lower in France than in Germany, Australia, and South Africa, while in South Africa it is lower than in Germany. Social well-being of respondents is relatively high and similar in Australia and South Africa and higher in these countries when compared to Germany and France. Functional well-being of survey respondents is higher in Australia than in the other countries, with Germany, South Africa, and France following in descending order. Finally, financial well-being in terms of expected future financial security is lowest amongst survey respondents from France compared to those from both Australia and South Africa, while financial well-being in terms of current money management stress is lowest amongst survey respondents in South Africa compared to those from both France and Australia.

### Correlations between the different dimensions of individual well-being

Table 6 presents correlations between the dimensions of well-being across and within countries for the survey respondents. We find that in the overall sample, all dimensions of well-being are correlated with each other, with financial well-being in terms of current money management stress being negatively correlated to individuals’ mental, social, functional, and financial well-being in terms of expected future financial security. When looking at individual countries, we observe some interesting differences, with functional well-being only being positively correlated with social well-being in France but not in the other countries.

**Table 6:** Correlations between the different dimensions of survey respondents’ well-being during the COVID-19 pandemic.

**A. All countries**				
	**(1)**	**(2)**	**(3)**	**(4)**
**(1) Mental Well-Being**				
**(2) Social Well-Being**	.450***			
**(3) Functional Well-Being**	.275***	.066***		
**(4) Financial Well-Being: Expected Financial Security**	.428***	.452***	.118***	
**(5) Financial Well-Being: Current Money Management Stress**	-.310***	-.070*	-.177***	-.387***
**B. Australia**				
	**(1)**	**(2)**	**(3)**	**(4)**
**(1) Mental Well-Being**				
**(2) Social Well-Being**	.537***			
**(3) Functional Well-Being**	.269***	-.075		
**(4) Financial Well-Being: Expected Financial Security**	.470***	.474***	.113*	
**(5) Financial Well-Being: Current Money Management Stress**	-.425***	-.200***	-.365***	-.486***
**C. France**				
	**(1)**	**(2)**	**(3)**	**(4)**
**(1) Mental Well-Being**				
**(2) Social Well-Being**	.364***			
**(3) Functional Well-Being**	.335***	.088*		
**(4) Financial Well-Being: Expected Financial Security**	.380***	.365***	.135**	
**(5) Financial Well-Being: Current Money Management Stress**	-.326***	-.137***	-.096*	-.494***
**D. Germany**				
	**(1)**	**(2)**	**(3)**	**(4)**
**(1) Mental Well-Being**				
**(2) Social Well-Being**	.382***			
**(3) Functional Well-Being**	.329***	.050		
**(4) Financial Well-Being: Expected Financial Security**	.330***	.412***	.050	
**(5) Financial Well-Being: Current Money Management Stress**	-.351***	-.037	-.171***	-.365***
**E. South Africa**				
	**(1)**	**(2)**	**(3)**	**(4)**
**(1) Mental Well-Being**				
**(2) Social Well-Being**	.489***			
**(3) Functional Well-Being**	.149***	-.008		
**(4) Financial Well-Being: Expected Financial Security**	.492***	.503***	.098*	
**(5) Financial Well-Being: Current Money Management Stress**	-.220***	-.109*	-.217***	-.329***

Correlation coefficients at level of significance of ***p < .001, **p < .010, *p < .050 (two-tailed). N = 2,100.

Correlation coefficients at level of significance of ***p < .001, **p < .010, *p < .050 (two-tailed). *n* = 494.

Correlation coefficients at level of significance of ***p < .001, **p < .010, *p < .050 (two-tailed). n = 549.

Correlation coefficients at level of significance of ***p < .001, **p < .010, *p < .050 (two-tailed). n = 510.

Correlation coefficients at level of significance of ***p < .001, **p < .010, *p < .050 (two-tailed). *n* = 547.

### Within- and across-country drivers of individual well-being

To assess the drivers of individual well-being of survey respondents, we carry out linear regressions for each dimension of well-being for each of the four studied countries as well as across the sample as a whole. Our regression set-up in terms of analyzing drivers across the entire sample as well as within each individual country sample follows the recent approach taken by Dryhurst et al. [21].

Starting with mental well-being (Table 7A), we find that it is associated with a direct COVID-19 experience only in Australia. Mental well-being is higher among older (except for South Africa), employed (in Australia and France), and male individuals (in France). Being part of an ethnic minority (i.e., non-White in Australia, France, and Germany; non-Black in South Africa) has a different effect across the countries: while it is associated with higher mental well-being amongst survey respondents in France, it is related to a lower level of well-being amongst survey respondents in South Africa. Only a few of the objective indicators of living conditions have a significant effect on mental well-being and only in specific countries (i.e., the presence of an external space is positively related to mental well-being amongst survey respondents in France and living in the city is positively related to well-being in the overall sample). However, the subjective evaluation of these conditions is positively and significantly related to individuals’ mental well-being across all the country samples. Positive health behaviors are positively associated with mental well-being of survey respondents in every country but Germany, while positive financial behaviors are positively associated with mental well-being of survey respondents in Germany and South Africa only.

**Table 7:** Regressions explaining the drivers of survey respondents’ well-being during the COVID-19 pandemic.

**A. Mental Well-Being**					
**Variables**	**All countries**	**Australia**	**France**	**Germany**	**South Africa**
	***B (SE)***	***B (SE)***	***B (SE)***	***B (SE)***	***B (SE)***
**COVID-19 Experience**	-.048 (.052)	-.272*(.011)	.058 (.095)	-.041 (.104)	-.066 (.102)
**Socio-Demographics**					
Age	.007***(.002)	.011**(.003)	.011***(.003)	.014***(.003)	.004 (.004)
Education	-.059 (.040)	-.054(.076)	-.047 (.072)	.032 (.082)	-.071 (.101)
Employment (working)	.205***(.056)	.272*(.111)	.354***(.102)	.066 (.106)	.073 (.127)
Ethnicity (minority)	-.183**(.060)	-.054 (.124)	.340*(.175)	.294 (.176)	-.617***(.120)
Gender (female)	-.250***(.049)	-.120 (.103)	-.405***(.088)	-.076 (.092)	-.191 (.106)
Income	-.022 (.021)	.032 (.037)	-.021 (.038)	.021 (.040)	.067 (.057)
**Objective Indicators of Living Conditions During Lockdowns**					
Area of residence (urban)	.186**(.060)	.151 (.151)	.064 (.093)	.112 (.105)	.025 (.178)
External space (presence)	.149 (.080)	.019 (.190)	.334*(.146)	.228 (.148)	-.006 (.154)
Number of dependents in household	.023 (.019)	.067 (.042)	.043 (.037)	.010 (.046)	-.014 (.032)
Size of indoor space (m^2^)	-.001 (.017)	.041 (.028)	-.069 (.039)	-.034 (.041)	-.016 (.030)
Relationship status (in couple)	.022 (.055)	-.002 (.106)	-.014 (.107)	-.093 (.110)	.215 (.113)
**Subjective Evaluation of Living Conditions During Lockdowns**	.180*** (.016)	.219***(.041)	.244***(.031)	.205***(.028)	.183***(.034)
**Positive Health Behaviors (during COVID-19)**	.123***(.016)	.195***(.031)	.138***(.031)	.034 (.030)	.186***(.035)
**Positive Financial Behaviors (during COVID-19)**	.070***(.015)	.046 (.028)	.051 (.034)	.092**(.030)	.096***(.030)
**Adjusted R^2^**	.185	.272	.237	.197	.244
***N***	2,100	494	549	510	547
***F***	*F*(15, 2084) = 32.765***	*F*(15, 478) = 13.256***	*F*(15, 533) = 12.360***	*F*(15, 494) = 9.320***	*F*(15, 531) = 12.734***
**B. Social Well-Being**					
**Variables**	**All countries**	**Australia**	**France**	**Germany**	**South Africa**
	***B (SE)***	***B (SE)***	***B (SE)***	***B (SE)***	***B (SE)***
**COVID-19 Experience**	-.007 (.056)	-.111 (.124)	-.058 (.103)	-.056 (.111)	.091 (.097)
**Socio-Demographics**					
Age	-.006**(.002)	.005 (.004)	.000 (.004)	-.004 (.004)	.002 (.004)
Education	.104*(.043)	.185*(.085)	.113 (.078)	.011 (.078)	.047 (.096)
Employment (working)	.184**(.060)	.230 (.125)	.329**(.111)	-.136 (.114)	.227 (.120)
Ethnicity (minority)	-.117 (.065)	.174 (.140)	.470*(.189)	.400*(.189)	-1.003***(.114)
Gender (female)	-.197***(.053)	.056 (.116)	-.267**(.096)	-.243*(.098)	-.036 (.101)
Income	.012 (.022)	.018 (.042)	-.020 (.041)	.129**(.043)	.088 (.054)
**Objective Indicators of Living Conditions During Lockdowns**					
Area of residence (urban)	.321***(.065)	.014 (.170)	.153 (.101)	.073 (.112)	-.050 (.169)
External space (presence)	.327***(.086)	.040 (.214)	.469**(.158)	.432**(.158)	.251 (.146)
Number of dependents in household	.083***(.020)	.113*(.047)	.070 (.040)	.100*(.049)	.041 (.030)
Size of indoor space (m^2^)	.007 (.018)	.030 (.032)	-.059 (.042)	-.053 (.044)	-.028 (.029)
Relationship status (in couple)	.012 (.059)	.036 (.119)	-.033 (.116)	-.097 (.118)	.207(.107)
**Subjective Evaluation of Living Conditions During Lockdowns**	.123***(.017)	.150***(.046)	.080*(.033)	.146***(.030)	.104***(.032)
**Positive Health Behaviors (during COVID-19)**	.177***(.017)	.184***(.035)	.181***(.033)	.127***(.032)	.272***(.033)
**Positive Financial Behaviors (during COVID-19)**	.012 (.016)	.017 (.032)	.062(.036)	.028 (.032)	.024 (.028)
**Adjusted R^2^**	.159	.156	.148	.151	.347
***N***	2,100	494	549	510	547
***F***	*F*(15, 2084) = 27.369***	*F*(15, 478) = 7.055***	*F*(15, 533) = 7.323***	*F*(15,494) = 7.024***	*F*(15, 531) = 20.314***
**C. Functional Well-Being**					
**Variables**	**All countries**	**Australia**	**France**	**Germany**	**South Africa**
	***B (SE)***	***B (SE)***	***B (SE)***	***B (SE)***	***B (SE)***
**COVID-19 Experience**	-.112 (.061)	-.188 (.123)	-.079 (.122)	.070 (.137)	-.013 (.100)
**Socio-Demographics**					
Age	.006**(.002)	.012***(.004)	.000 (.004)	.007 (.004)	.003 (.004)
Education	-.285***(.047)	-.137 (.084)	-.320***(.091)	-.250**(.096)	-.183 (.099)
Employment (working)	.005 (.065)	-.136 (.123)	.038 (.130)	-.095 (.140)	.064 (.124)
Ethnicity (minority)	.033 (.070)	-.167 (.138)	.112 (.223)	-.007 (.232)	.126 (.118)
Gender (female)	-.111*(.057)	.027 (.115)	-.143 (.112)	-.058 (.120)	.020 (.104)
Income	-.078***(.024)	.003 (.042)	-.088 (.049)	.010 (.040)	.047 (.056)
**Objective Indicators of Living Conditions During Lockdowns**					
Area of residence (urban)	.038 (.070)	-.196 (.168)	-.246*(.119)	-.063 (.138)	-.098 (.174)
External space (presence)	-.013 (.093)	-.141 (.212)	.177 (.186)	-.339 (.194)	.064 (.150)
Number of dependents in household	-.057**(.022)	-.065 (.046)	-.016 (.047)	.011 (.060)	-.073*(.031)
Size of indoor space (m^2^)	.012 (.020)	-.016 (.031)	-.031 (.049)	-.005 (.054)	-.014 (.030)
Relationship status (in couple)	-.012 (.065)	-.019 (.118)	.042 (.136)	-.066 (.145)	.018 (.110)
**Subjective Evaluation of Living Conditions During Lockdowns**	.272***(.018)	.194***(.045)	.271***(.039)	.383***(.037)	.085**(.033)
**Positive Health Behaviors (during COVID-19)**	.020 (.019)	.019 (.034)	.125***(.039)	-.012 (.040)	.004 (.034)
**Positive Financial Behaviors (during COVID-19)**	.020 (.018)	.097**(.032)	-.077 (.043)	.010 (.040)	.085**(.029)
**Adjusted R^2^**	.143	.148	.132	.219	.051
***N***	2,100	494	549	510	547
***F***	*F*(15,2084) = 24.328***	*F*(15,478) = 6.698***	*F*(15,533) = 6.534***	*F*(15,494) = 10.505***	*F*(15,531) = 2.937***
**D. Financial Well-Being: Expected Financial Security**					
**Variables**	**All countries**	**Australia**	**France**	**Germany**	**South Africa**
	***B (SE)***	***B (SE)***	***B (SE)***	***B (SE)***	***B (SE)***
**COVID-19 Experience**	.063 (.064)	-.089 (.145)	.176 (.118)	-.093 (.131)	.172 (.117)
**Socio-Demographics**					
Age	-.011***(.002)	-.002 (.005)	.002 (.004)	-.006 (.004)	-.026*** (.004)
Education	.188***(.049)	.300**(.099)	.126 (.089)	.194 (.092)	.271*(.116)
Employment (working)	.136*(.068)	.170 (.146)	.110 (.126)	.193 (.134)	.175 (.145)
Ethnicity (minority)	-.494***(.074)	-.135 (.163)	-.128 (.216)	.238 (.222)	-.793***(.138)
Gender (female)	-.224***(.060)	-.318*(.135)	-.287**(.108)	-.060 (.115)	-.115 (.122)
Income	.196***(.026)	.151**(.049)	.274***(.047)	.288***(.050)	.234***(.066)
**Objective Indicators of Living Conditions During Lockdowns**					
Area of residence (urban)	.204**(.074)	-.134 (.198)	.104 (.116)	.013 (.132)	.175 (.204)
External space (presence)	.270**(.098)	.088 (.250)	.268 (.181)	.311 (.186)	.293 (.177)
Number of dependents in household	-.012 (.023)	.009 (.055)	.023 (.045)	.024 (.058)	-.031 (.036)
Size of indoor space (m^2^)	.038 (.021)	.047 (.037)	-.025 (.048)	.005 (.051)	.007 (.035)
Relationship status (in couple)	.045 (.068)	.230 (.139)	-.187 (.132)	-.068 (.138)	.125 (.129)
**Subjective Evaluation of Living Conditions During Lockdowns**	.145***(.019)	.166**(.053)	.172***(.038)	.155***(.136)	.142***(.039)
**Positive Health Behaviors (during COVID-19)**	.143***(.020)	.174***(.040)	.189***(.038)	.104**(.038)	.156***(.040)
**Positive Financial Behaviors (during COVID-19)**	.224***(.019)	.242***(.037)	.256***(.042)	.255***(.038)	.202***(.034)
**Adjusted R^2^**	.302	.308	.330	.358	.381
***N***	2,100	494	549	510	547
***F***	*F*(15,2084) = 61.508***	*F*(15,478) = 15.645***	*F*(15,533) = 19.017***	*F*(15,494) = 19.923***	*F*(15,531) = 23.440***
**E. Financial Well-Being: Current Money Management Stress**					
**Variables**	**All countries**	**Australia**	**France**	**Germany**	**South Africa**
	***B (SE)***	***B (SE)***	***B (SE)***	***B (SE)***	***B (SE)***
**COVID-19 Experience**	.150*(.061)	.264 (.134)	-.016 (.105)	.147 (.135)	.231*(.118)
**Socio-Demographics**					
Age	-.015***(.002)	-.020***(.004)	-.010**(.004)	-.021***(.004)	-.001 (.004)
Education	-.051 (.047)	-.007 (.092)	-.129 (.079)	-.180 (.095)	.045 (.116)
Employment (working)	.168 (.065)	.286*(.134)	-.006 (.113)	.260 (.139)	-.008 (.146)
Ethnicity (minority)	.102 (.071)	-.093 (.150)	-.217 (.193)	.076 (.229)	-.202 (.139)
Gender (female)	.026 (.058)	-.024 (.125)	.012 (.097)	-.099 (.119)	.050 (.122)
Income	-.088***(.024)	-.080 (.045)	-.114 (.042)	-.184***(.052)	-.018 (.
**Objective Indicators of Living Conditions During Lockdowns**					
Area of residence (urban)	-.027 (.071)	-.371*(.182)	-.076 (.103)	-.058 (.136)	-.077 (.205)
External space (presence)	-.017 (.094)	-.180 (.230)	.091 (.161)	.036 (.192)	-.077 (.177)
Number of dependents in household	.091***(.022)	.118 (.051)	.091*(.040)	.054 (.060)	.095**(.036)
Size of indoor space (m^2^)	.022 (.020)	-.011 (.034)	.016 (.042)	.068 (.053)	.020 (.035)
Relationship status (in couple)	.088 (.065)	-.100 (.128)	.016 (.118)	.261 (.143)	.302*(.130
**Subjective Evaluation of Living Conditions During Lockdowns**	-.068***(.018)	-.236***(.049)	-.183***(.034)	-.094*(.037)	-.030 (.039)
**Positive Health Behaviors (during COVID-19)**	-.039*(.019)	-.111**(.037)	-.026 (.034)	.057 (.039)	-.078 (.040)
**Positive Financial Behaviors (during COVID-19)**	-.282***(.018)	-.263***(.034)	-.205***(.037)	-.284***(.039)	-.299***(.035)
**Adjusted R^2^**	.247	.360	.213	.250	.192
***N***	2,100	494	549	510	547
***F***	*F*(15,2084) = 46.936***	*F*(15,478) = 19.510***	*F*(15,533) = 10.905***	*F*(15,494) = 12.314***	*F*(15,531) = 9.639***

*** p < .001; ** p < .010; * p < .050. SE = standard error. Statistically significant effects in bold. Education: 1 = incomplete secondary; 2 = complete secondary; 3 = complete tertiary. Employment: not working = unemployed, retired, or other; working = employed or self-employed. Ethnicity: minority = non-white in Australia, France, and Germany; non-black in South Africa. Relationship: not in a relationship = single, divorced, or widowed; in a relationship = married, civil union, permanent partner/de-facto.

*** p < .001; ** p < .010; * p < .050. SE = standard error. Statistically significant effects in bold. Education: 1 = incomplete secondary; 2 = complete secondary; 3 = complete tertiary. Employment: not working = unemployed, retired, or other; working = employed or self-employed. Ethnicity: minority = non-white in Australia, France, and Germany; non-black in South Africa. Relationship: not in a relationship = single, divorced, or widowed; in a relationship = married, civil union, permanent partner/de-facto.

*** p < .001; ** p < .010; * p < .050. SE = standard error. Statistically significant effects in bold. Education: 1 = incomplete secondary; 2 = complete secondary; 3 = complete tertiary. Employment: not working = unemployed, retired, or other; working = employed or self-employed. Ethnicity: minority = non-white in Australia, France, and Germany; non-black in South Africa. Relationship: not in a relationship = single, divorced, or widowed; in a relationship = married, civil union, permanent partner/de-facto.

*** p < .001; ** p < .010; * p < .050. SE = standard error. Statistically significant effects in bold. Education: 1 = incomplete secondary; 2 = complete secondary; 3 = complete tertiary. Employment: not working = unemployed, retired, or other; working = employed or self-employed. Ethnicity: minority = non-white in Australia, France, and Germany; non-black in South Africa. Relationship: not in a relationship = single, divorced, or widowed; in a relationship = married, civil union, permanent partner/de-facto.

*** p < .001; ** p < .010; * p < .050. SE = standard error. Statistically significant effects in bold. Education: 1 = incomplete secondary; 2 = complete secondary; 3 = complete tertiary. Employment: not working = unemployed, retired, or other; working = employed or self-employed. Ethnicity: minority = non-white in Australia, France, and Germany; non-black in South Africa. Relationship: not in a relationship = single, divorced, or widowed; in a relationship = married, civil union, permanent partner/de-facto.

Next, we examine the drivers of social well-being (Table 7B) and find that being more educated (Australia and overall sample), employed (France and overall sample), male (France, Germany, and overall sample), and having higher income (Germany) contributes to higher social well-being. Again, belonging to an ethnic minority has a positive effect amongst survey respondents in France and Germany and a negative effect amongst survey respondents in South Africa. Area of residence (overall sample), presence of external space (overall sample, France, and Germany), and number of dependents in household (overall sample and France) are positively associated to social well-being. Also, the subjective evaluation of one’s living conditions and positive health behaviors are positively associated with social well-being in the overall sample.

Functional well-being is higher for older (Australia and overall sample), less educated (France and Germany), male (overall sample), and lower-income people (overall sample) (Table 7C). Individuals with a large number of dependents living in their household (South Africa) have lower functional well-being. We also find that one’s subjective evaluation of living conditions is positively associated with functional well-being in the overall sample, while positive health behaviors are positively related to functional well-being amongst survey respondents in France, and positive financial behaviors are positively associated with functional well-being amongst survey respondents in Australia and South Africa.

Finally, amongst our survey respondents, being younger (South Africa and overall sample), better educated (Australia, South Africa, and overall sample), employed (overall sample), male (Australia and France), having a higher income, and being non-White in South Africa is positively associated with financial well-being in terms of expected future financial security (Table 7D). At the same time, being older (all countries except in South Africa), having higher income (in Germany), living in the city (in Australia), and having fewer dependents (in France) is negatively associated with financial well-being in terms of being associated with higher current money management stress (Table 7E). One’s subjective evaluation of living conditions during lockdowns is positively associated with financial well-being in terms of higher expected future financial security and lower current money management stress (except for South Africa). Similarly, positive financial behaviors are positively associated with financial well-being. Finally, positive health behaviors are positively and significantly associated with financial well-being in terms of higher expected future financial security amongst our survey respondents in every country, while it is only negatively associated with current money management stress for Australian respondents.

## Discussion

We expected that individuals’ mental, social, functional, and financial well-being are correlated during the pandemic. Our results indicate a multifaceted picture in this regard amongst survey respondents. Across all countries, the different dimensions of well-being are correlated, which supports our holistic approach in studying individual well-being. Notably, social well-being is strongly correlated with mental and financial well-being, confirming the importance of relational goals for overall well-being [58]. Although they are correlated in the overall sample including all countries, in line with prior research by Barrett, Hogreve [23], functional and social well-being are generally not correlated amongst survey respondents within individual countries, with those in France being an exception. Cultural differences (e.g., individuals’ preferences for certain types of social interaction), or differences in economic and sanitary contexts across the four countries may help explain such differences. For example, the general lack of a correlation between functional and social well-being could be explained by online interactions reducing loneliness during the pandemic [59] or by individual differences related to personality characteristics [60]. The different result for respondents in France could be explained by its culture leading to a higher desire for interactions beyond the household [61].

Overall, at the time of the survey, individual well-being is highest amongst survey respondents in Australia and lowest amongst survey respondents in France, confirming our expectation of cross-country differences along the individual well-being dimensions during the pandemic. These cross-country differences in individual well-being might, in part, be explained by differences in the health and economic situation in the four countries, and the policy measures of their respective governments at the time of our survey [23]. Accordingly, Australia was in the best position with closed borders and low case numbers and low death rate, while South Africa benefitted from the lowest “alert Level 1” before the third wave struck both countries. Germany started to ease its restrictions during our sample period, while France only planned for a gradual return to normal life towards the end of our sample period. See Table 4 for details.

Our results suggest that strict public health restrictions such as in France are associated with a lower level of well-being. Indeed, at the time of our survey in May 2021, the French had been in lockdown for over 125 days, enduring working and schooling from home, facing restrictions to leave their home, and being unable to visit relatives living in a different home. This might help explain why overall well-being is lowest amongst survey respondents in France. In contrast, up to and including the time of data collection, Australia benefited from a minimum of restrictions, which could help explain the higher levels of social and functional well-being amongst survey respondents in this country. Furthermore, its low number of COVID-19 cases and low death rate could have had a reassuring effect on individuals. Together with the access to specific COVID-19 mental health support from early 2021, these contextual features could help explain the comparably high level of mental well-being amongst survey respondents in Australia during the sample period.

Throughout the sample period, Germany experienced the highest within-country increase in COVID-19 cases and death rate. We expect the perceived threat of this situation to individuals to be associated with lower mental well-being [10]. South African respondents report comparably high overall well-being, however. We find that South Africa is the only country in which there is a positive correlation between functional well-being and financial well-being in terms of expected future financial security, which could be explained by the weaker financial support packages for people affected by COVID-19 in this country. That is, without financial support, if individuals are restricted in their mobility and ability to go about their daily activities, this jeopardizes their expected future financial security.

Our expectation of a negative association between a direct COVID-19 experience and well-being during the pandemic is not confirmed, with the exception of mental well-being in Australia and current money management stress in South Africa. However, we find ample and again multifaceted support for our expectations that socio-demographic factors and objective living conditions are associated with differences in individual well-being during the pandemic. Interestingly, our results suggest that COVID-19 and the related lockdowns and public health restrictions were easier to cope with mentally and socially in terms of their association with individual well-being amongst survey respondents for older people and people from non-white ethnicities (i.e., in France, Germany, and South Africa). The former result might be explained by the fact that compared to older individuals, younger individuals have been shown to have higher levels of anxiety and overeating compensatory behavior during the pandemic [30]. Moreover, these results are in line with prior findings that young adults feel more constrained and less autonomous due to social and movement restrictions during the pandemic [62]. Indeed, while older individuals were more concerned by COVID-19 in the very beginning of the epidemic, they were less worried about the virus during the lockdowns and changed their behavior to a lesser extent [63]. The latter result might be explained by prior studies that find that ethnic minorities have been more impacted by COVID-19 [64], have lower vaccination levels and intentions [65], and lower levels of mental well-being [66]. On the other hand, ethnic minorities usually leave in bigger families [64], which might explain less loneliness, higher social well-being, and consequently higher mental well-being in our sample.

Another surprising finding is the effect of education on the different dimensions of well-being. In particular, amongst our survey respondents, education is positively associated with social well-being and financial well-being in terms of expected future financial security (in Australia) and is negatively associated with functional well-being (in France and in Germany). Our findings build on prior research which finds that higher education is generally positively associated with physical and mental well-being [67]. Furthermore, in Australia, higher education is associated with higher incomes and employment [68]. These income and employment advantages of better educated Australians is reflected in their higher financial well-being. At the same time, more highly educated individuals require better conditions in order to be satisfied with their well-being [69], which could help explain the negative association between education and functional well-being in France and Germany. In particular, individuals with higher levels of education tend to engage more in outdoor and sportive leisure activities as they search to reduce stress and counterbalance their sedentary working lives [70], which they were unable to do due to the public health restrictions associated with the pandemic. This explanation is in line with findings by Belo, Navarro-Pardo [71] on how leisure attitude mediates the link between education level and well-being.

Although socio-demographic factors and objective living conditions during lockdowns matter in different countries and across different well-being dimensions, the subjective evaluation of one’s living conditions during lockdowns is associated with reported levels of individual well-being amongst our survey respondents in every country in our sample and across all well-being dimensions except for current money management stress in South Africa. This strongly confirms our expectation that the subjective evaluation of one’s living conditions is associated with individual well-being during the pandemic and is in line with prior studies highlighting the impact of residential well-being on other dimensions of perceived well-being [36, 39].

Finally, we find strong support for our last expectation regarding the importance of positive health and financial behaviors in terms of their association with the different dimensions of individual well-being. Notably, positive health behaviors are positively associated with social and financial well-being, while positive financial behaviors are not only associated with higher levels of financial well-being, but are also associated with higher levels of mental, social, and functional well-being in some of the countries. These results build on prior research finding that exercising during COVID-19 boosts one’s mental as well as physical well-being [17]. Moreover, generally, positive financial behaviors are related to higher levels of financial well-being [25]. Importantly, extending Hoffmann and Risse [6], we also find that positive financial and health behaviors spill over onto seemingly unrelated dimensions of individual well-being. For example, positive health behaviors are also positively associated with financial well-being while positive financial behaviors are also positively associated with mental, social, and functional well-being.

In sum, despite the necessity of health restrictions to reduce the spread of the COVID-19 virus, we find that in countries where those restrictions are stricter, individual well-being is lower. Although establishing causality is beyond the scope of this study, our initial evidence suggests policymakers should apply lockdown restrictions with prudence [72] and take into account their potential deteriorating effect on individuals’ overall well-being [cf. 23].

## Conclusion

Although individuals’ physical well-being might be safeguarded through strict health restrictions, their mental, functional, and financial well-being are likely to suffer. Importantly, the interrelation between these different dimensions of individual well-being should be considered, to prevent gains in one dimension being offset by losses in another. Our results indicate that it is important to address the link between mental well-being and financial well-being in terms of current money management stress. We thus suggest a continuous investment in mental health programs, such as the ongoing “Head to Health” initiative in Australia. Our results also support the importance of timely financial support, such as disaster payments and unemployment benefits to try and alleviate impairments of financial well-being and the associated spill-over effects into other dimensions of well-being [6]. With regards to the importance of positive health and financial behaviors for the different dimensions of individual well-being, we suggest launching and supporting free versions of programs and apps aiming at installing and keeping positive habits, such as Noom (for healthy eating), Nike Training Coach (for sport exercises), PocketGuard Best for Overspenders (for budgeting and spending), or Grow Habit Tracking (for setting and sticking to good habits). Similarly, such campaigns as #HealthyAtHome by the WHO are important for overall individual well-being during and after lockdowns.

We acknowledge that our cross-sectional data does not allow us to establish causality, and recommend future studies to use longitudinal data to unravel the causal nature of the relationship between the drivers of well-being and individuals’ scores on the different dimensions of well-being.

Another limitation of our current study is that we ask respondents in 2021 about their historical COVID-19 experience from February 2020 onwards, which might suffer from recall bias. Finally, the set of explanatory variables included in our analyses is not exhaustive, and future studies could explore the effect of other contextual factors, such as having sports facilities at home, having a pet that requires an active lifestyle, having a good relationship with one’s other household members, and working from home full-time, on differences in individual well-being.

Despite its limitations, our study contributes to the emerging literature on individual well-being during the COVID-19 pandemic [e.g., 5, 60, 73]. In particular, we extend prior literature by providing a holistic overview of the variation in and correlation between the different dimensions of individual well-being (i.e., mental, social, functional, and financial) during the pandemic and their drivers across four different countries as well as the role of a direct COVID-19 experience [cf. 21].

## Supporting information

S1 Data (SAV)
